# An Intelligent Content Discovery Technique for Health Portal Content Management

**DOI:** 10.2196/medinform.2671

**Published:** 2014-04-23

**Authors:** Daswin De Silva, Frada Burstein

**Affiliations:** ^1^Centre for Organisational and Social InformaticsFaculty of ITMonash UniversityMelbourneAustralia

**Keywords:** health information retrieval, personalised content management, health information portal, fuzzy multi-criteria ranking, automated content discovery, data analytics, text mining

## Abstract

**Background:**

Continuous content management of health information portals is a feature vital for its sustainability and widespread acceptance. Knowledge and experience of a domain expert is essential for content management in the health domain. The rate of generation of online health resources is exponential and thereby manual examination for relevance to a specific topic and audience is a formidable challenge for domain experts. Intelligent content discovery for effective content management is a less researched topic. An existing expert-endorsed content repository can provide the necessary leverage to automatically identify relevant resources and evaluate qualitative metrics.

**Objective:**

This paper reports on the design research towards an intelligent technique for automated content discovery and ranking for health information portals. The proposed technique aims to improve efficiency of the current mostly manual process of portal content management by utilising an existing expert-endorsed content repository as a supporting base and a benchmark to evaluate the suitability of new content

**Methods:**

A model for content management was established based on a field study of potential users. The proposed technique is integral to this content management model and executes in several phases (ie, query construction, content search, text analytics and fuzzy multi-criteria ranking). The construction of multi-dimensional search queries with input from Wordnet, the use of multi-word and single-word terms as representative semantics for text analytics and the use of fuzzy multi-criteria ranking for subjective evaluation of quality metrics are original contributions reported in this paper.

**Results:**

The feasibility of the proposed technique was examined with experiments conducted on an actual health information portal, the BCKOnline portal. Both intermediary and final results generated by the technique are presented in the paper and these help to establish benefits of the technique and its contribution towards effective content management.

**Conclusions:**

The prevalence of large numbers of online health resources is a key obstacle for domain experts involved in content management of health information portals and websites. The proposed technique has proven successful at search and identification of resources and the measurement of their relevance. It can be used to support the domain expert in content management and thereby ensure the health portal is up-to-date and current.

## Introduction

### Background

The Internet has become a key medium for audiences seeking health information resources [1]; an important contributor is health information portals. Content management (CM) in health information portals covers a broad spectrum of functions that surround the creation, discovery, distribution, consumption, and maintenance of content. A mixture of cyclic and acyclic execution of these functions is evident in both research and industrial applications. Large organizations usually follow the full cycle from content creation to maintenance, whereas specific applications focus on the advancement of a limited number of functions. Each function has its own challenges with added complexity introduced by the context of the application.

CM is a widely published topic with research conducted in knowledge management [2], Internet research [3], and information retrieval [4]. The focus of research in CM is largely influenced by its context. This context varies from enterprise level management to management of basic website content. At the enterprise level, recent advances include the ECM3 model [5], which aims to address the CM challenges by introducing stages of maturity for all enterprise documents and unstructured content. The Web content maturity model proposed by Forrester research [6] attempts to address the challenges facing an organization’s Web content. It consists of 4 phases: basic, tactical, enterprise, and engagement. The focus gradually broadens through these 4 phases, starting with the basic focus of making enterprise content available online and in the final phase expanding it to providing an online channel to achieve organizational goals. The *Content Management Bible* [7] defines CM as composed of 3 phases: the first is creation or collection of content; the second is managing storage and retrieval, versioning over time, and multiple languages etc; and the third involves publication and delivery of the content.

Content discovery plays an important part in CM as a quality intensive function that also determines the level of acceptance by a target audience. For instance, low quality and irrelevant content that fails to gain attention would limit the usefulness of the entire CM process. The significance of content discovery is also evident through its contribution to a broad spectrum of technologies, including portals (enterprise, information, and community), wikis, e-commerce, and social media.

Domain expertise is integral to content discovery. The domain expert needs to be proficient in both the subject area as well as the process of acquiring content relevant to a well-defined audience. A domain expert would maintain a high degree of emphasis on the quality of content as well as the level of personalization. Quality is generally identified in terms of 4 factors: relevance, usefulness, reliability, and timeliness [8]. Personalization addresses the diverse interests, needs, and expectations of a target audience composed of several subgroups [9].

Domain experts involved in content discovery for health information portals are confronted with an exponential growth in online content. Although access to most content is simplified by the availability of search engines, the discovery of relevant, high quality content that is personalized to suit the information needs of a target audience remains a challenge. In this paper, we propose an intelligent content discovery technique to address the challenge. This paper follows the design science research process to solve this important real world problem by designing a solution (information technology artefact) in a form of an innovative automated content discovery and ranking approach for health information portals [10].

The groundwork of the technique was reported in a previous publication [11]. The technique is based on the appropriation of an existing expert-endorsed content base as a benchmark to evaluate new content with similar features and offer the new content for inclusion to the portal repository. This semi-automated technique augments the manual process of content discovery, thus addressing inefficiencies, saving human effort, and potentially reducing human error with the increasing availability of online health information.

As stated, content discovery is relevant to a wide spectrum of technologies and application areas. This paper explores content discovery in the context of smart health information portals (SHIPs).

### Smart Health Information Portals

An information portal, in general, is a gateway to a diverse collection of information on a specific domain of interest. It attempts to aggregate information from multiple sources and present it in a useful form to targeted groups of users [12]. Advances in information systems coupled with the wide availability of diverse interfaces to the Internet have led to the adoption of smart technology for the development of portals. Within this context, it is pertinent to formally define a SHIP as the provision of smart technology and techniques to enhance the core capabilities of CM, content delivery, and collaboration for online health information provision [11]. The authors identify that it is not sufficient to define SHIP exclusively on its exhibiting computational intelligence features, for example, learning, reasoning, and memory. Sustainability of SHIP operation within organizational settings is crucial for its long-term viability. Hence, the issue of maintenance support becomes one of the deciding factors in the level of intelligence of a SHIP’s operation.

Breast Cancer Knowledge Online [13] and Heart Health Online [14] are examples of SHIPs researched and developed at the Faculty of Information Technology, Monash University, to address the health and medical information requirements of individuals associated with breast cancer, and mental health associated with heart conditions, including patients, caregivers, family, and friends of those affected. The delivery of user-sensitive, relevant, timely, and accurate health information to the various user groups was the focus throughout the various phases of the projects. These SHIPs are implementing several novel research outcomes, for example, resource description quality criteria modelling [15], user-centric portal design [16], automated quality assessment [8], and decision support systems perspective on portals [17]. Reported experience from the development of these SHIPs clearly demonstrated the value of continuous engagement and a high degree of reliance of user groups to identify, categorize, and describe the type of information required by relevant individuals. The resource intensity in terms of time and scarcity of relevant expertise was also highlighted by the researchers involved in these projects [17-19]. These studies reinforce the need for intelligent support for SHIP CM.

Automated content discovery, content summarization [20], dynamic ranking, user annotations, and feedback [21] are some of the enhancements to CM, which could assist in SHIP CM. Content delivery is enhanced with user profiling, geographical filtering, mobile interfaces, and device-independent content delivery. Online messaging, social networking, and discussion forums are enablers for smart collaboration. Among these features, assurance of quality of information delivery is by far the most sought after by users, and the most resource intensive from the organizational setup point of view.

### Content Management Model

The CM model represents the external entities of CM and their interactions in the formulation and management of personalized content. Informed by the experience with BCKOnline and Heart Health portal research [19], this model is a conceptualization of the fact that the audience of the SHIP users has distinct characteristics and contexts, which potentially affect their information needs. The resources for a SHIP can be aligned with a domain ontology, which classifies them against the major concepts that define such a domain. For example, official publications from medical journals are usually classified by a set of keywords, which the audience is likely to use to search and retrieve these publications. A set of such keywords or subject terms can be considered as part of domain ontology. The completeness or relevance of such an ontology can be problematic, especially when it comes to the search for relevant user-centered information [18]. It is up to the domain experts to reach consensus when deciding which terms are most suited for the ontology and content discovery. However, these issues are outside the scope of this particular paper. For this research we assume that there is a trusted and appropriate domain ontology constructed for resource classifications (eg, in BCKOnline, a combination of Medical Subject Headings [MeSH], BreastCare Victoria Glossary, BCKOnline Disease Trajectory, and BCKOnline keywords were used as encoding schemas for the subject metadata element [22]). The role of domain experts in classifying potential resources against the needs of the target audience becomes essential for identifying the best terminology suitable and understandable by the target audience.

**Figure 1:**
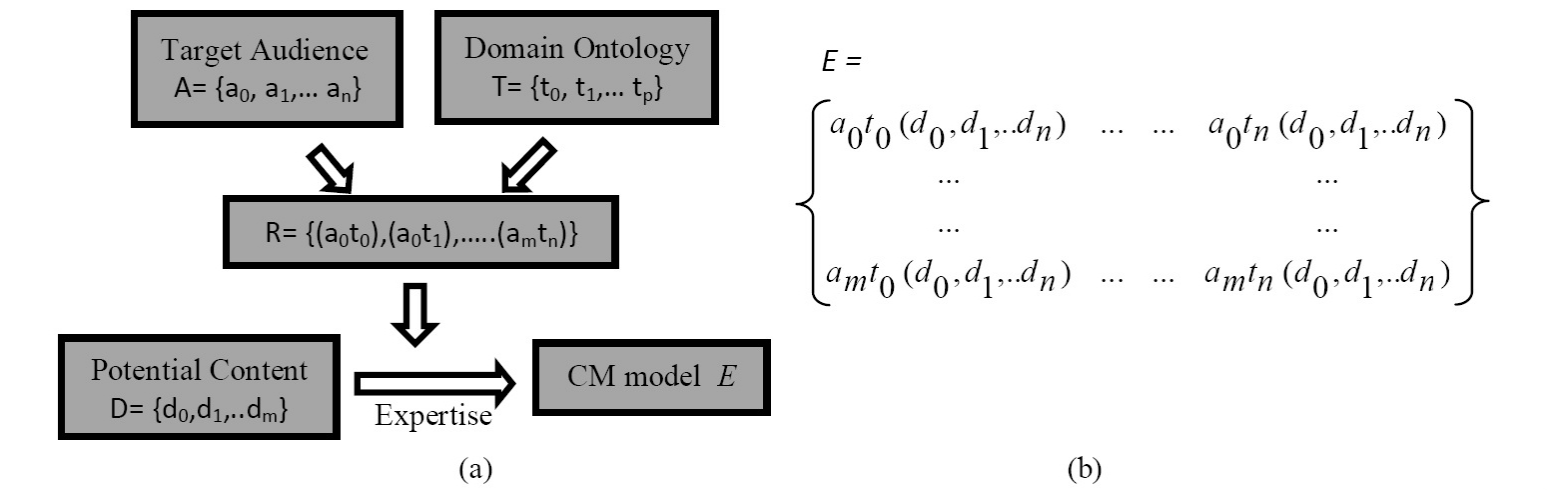
(a) Formulation of content management entities (b) SHIP content model as a matrix.

At the generic level, the target audience, potential content, a domain ontology, and domain expertise are the external entities that are fused together to generate personalized content. This formulation is further illustrated in Figure 1a. It is useful to formally define the entities and their interactions. The target audience comprises subgroups of users with similar characteristics and thus having similar information needs. Let *A={a*_*0*_ *, a*_*1*_ *,…a*_*n*_ *}* be the target audience comprising all subgroups. Let *D={d*_*0*_ *, d*_*1*_ *,…d*_*m*_ *}* be the set of all content that is able to address the information needs of the target audience. A domain ontology formalizes the concept hierarchy of knowledge for a specific domain, and it can be generally represented as a set of topics, *T={t*_*0*_ *, t*_*1*_ *,… t*_*p*_ *}.* The information requirements for audience *A* are determined using the Cartesian product of *A* and *T*. Let *R* be the Cartesian product, R=A*T. Actual information requirements could very well be a subset of *R* because all terms may not be applicable to all *A*. Domain expertise transforms information requirements *R*, to actual content *D*, by determining subsets of *D* that address each *R*. Let this transformation be *E={e*_*0*_ *, e*_*1*_ *,… e*_*x*_ *}* , where *e*_*0*_ *={a*_*0*_ *t*_*0*_ *,(d*_*0*_ *,d*_*1*_ *,…d*_*m*_ *)}* comprises information requirements and a set of matched content elements. The transformation *E* represents the CM model because it captures all entities and their relationships. It can also be depicted as a matrix (Figure 1b).

The CM model possesses certain properties that make it robust and flexible to changes. Over time, it is likely *A*, *T*, and *D* would expand or contract to reflect developments in health practices. Matrix *E* is time-invariant and thus can be altered easily to reflect these changes. The challenge and opportunity for developing a sustainable CM model is in designing transformation R as a semi-automated expert-driven procedure by using intelligent technologies. The following section elaborates on this technique.

## Methods

### Overview

**Figure 2:**
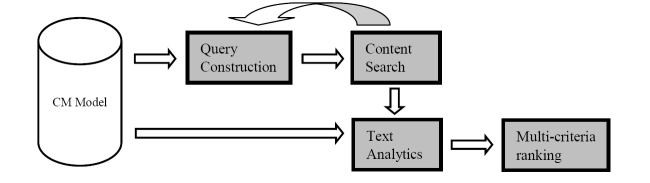
Proposed content discovery technique.

The CM model underlies the formulation of the proposed technique. It extracts semantics that are useful to construct queries that discover new content as well as semantics that are used to measure the relevance of new content from the CM model. Query construction introduces context specific information to the final query that is then distributed to search engines. The results are amalgamated and followed by the analysis of textual content of both new and existing resources. In the content selection phase, each item is ranked based on several factors of quality and presented to the domain expert for further perusal and possible inclusion in the content repository. Figure 2 illustrates the components of the technique.

### Query Construction and Content Search

**Figure 3:**
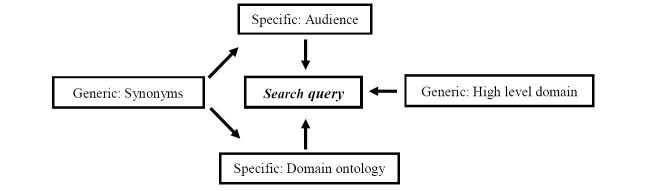
Elements in query construction.

Each query is based on several specific and generic dimensions. The specific dimensions are sourced from meta-data found in the first element of each term in the CM matrix (Figure 1b). The element *a*_*x*_ *t*_*y*_, denotes the audience subgrouping and the term (or topic) from the domain ontology. The generic dimensions serve the purpose of introducing the context/background to a search. These can range from the high-level domain terms to synonyms indicative of the specific dimensions. Figure 3 illustrates this further.

Both specific dimensions are well defined by the domain expert and thereby translate easily into query construction. The audience dimension will contain information about the subgroups found within. Age, sex, marital status, occupational status, and level of knowledge of the domain are some examples. The domain ontology contains the key terms and phrases that define the information needs of the audience. The generic dimension of synonyms introduces further diversity to the query construction process with related terms for the two specific dimensions. The widely used lexical database, WordNet [23] is used to extract synonyms with semantic relationships. WordNet is a lexical database for the English language. It is made up of two parts: sets of synonyms called (synsets) and the semantic relations between these sets. The semantic relations are useful to identify terms that have a common ancestor and thus can be linked to each other. For instance, wellness and well-being are terms similar in meaning to health but positioned at different levels on WordNet. Query construction will generate a set of queries *Q={q_1_,q_2_,….q_n_}*, representing the information needs expressed in the CM model.

Query construction and content search are recurrent phases in which queries with failed searches are reconstructed using synonyms from WordNet. In the content search phase, each query will be run on several search engines. Duplicates are removed from the search results generated and merged into one distinct set. The actual webpages are downloaded from this list and further examined for misrepresentations, such as duplicates, revisions of the same page, index pages, pages generated by other search engines, etc. The valid results are converted to plain text using Apache Tika, which is able to parse most Web document formats, including HTML, PDF, and XML. The resultant corpus of plain text documents, *D_q_=d_q1_, d_q2_, …d_qn_; ∀n∈N, ∀q∈Q*, is input to the text analytics phase.

### Text Analytics

#### Overview

**Figure 4:**
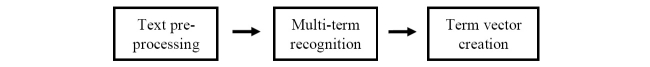
Text analytics sub-modules.

Text analytics is responsible for the identification of content that is relevant to the existing expert endorsed resources. It is the core function of the technique and is made up of 3 submodules as illustrated in Figure 4.

Text analytics is an emerging area in business analytics where smart techniques are being developed and used to extract patterns, predictions, and semantic content from text corpora [24]. Every document has a number of words used only for grammar and presentation and not directly related to content description. Preprocessing removes the words that do not have a semantic use for analysis. Stop-word removal [25] and Porter’s stemming algorithm [26] are run on the text corpus to generate a “bag of words” representation of each document. Further preprocessing can be conducted depending on the content of the original documents (formulae, images, and other media).

#### Multi-Term Recognition

**Figure 5:**
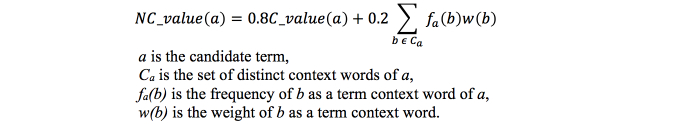
Calculation of NC-value.

Multi-term recognition aims to improve the semantic representation of the original document with the extraction of multi-word terms by means of the C-value/NC-value approach [27]. This method combines linguistic and statistical information with emphasis on nested multi-word terms and the general distribution of candidate terms. It has been used successfully in a variety of applications [28,29]. It generates a list of multi-word terms ranked by the NC-value. The NC-value is a weighted summation of context information and the C-value (Figure 5).

**Figure 6:**
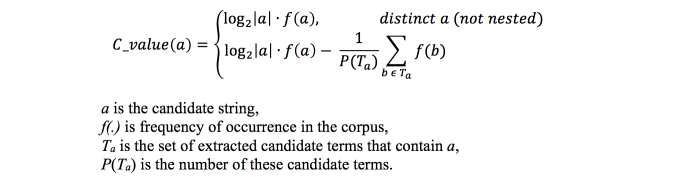
Calculation of C-value.

The 2 factors of NC-value have been assigned the weights 0.8 and 0.2, respectively, based on previous experiments [27]. The C-value is a measure of each term’s distinct frequency of occurrence within the corpus. It takes into account the number of times the term appears nested within other candidate terms; this is subtracted from the total frequency in the corpus (Figure 6).

**Figure 7:**
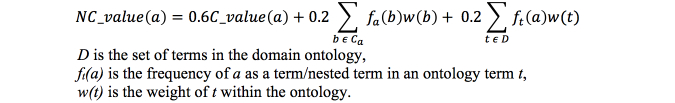
Calculation of NC-value with introduction of domain-specific information.

To improve the detection of multi-word terms, the C-value/NC-value approach was extended with the introduction of domain-specific information to the calculation of NC-value. The presence/absence of terms from the domain ontology was incorporated as shown in Figure 7.

The domain ontology is composed of terms recommended by the experts and thus would appropriately narrate the context of the search to each document. The new element in the equation captures the likelihood of candidate terms appearing within the domain ontology as nested or distinct terms. The weight of term *t* can be determined by the hierarchical organisation or its relationships within the ontology. The factors of the new NC-value have been assigned weights 0.6, 0.2, and 0.2, respectively This adjustment ensures that context factor and ontology information have equal contribution toward the final measure.

#### Term Vector Creation

The third submodule, term vector creation, generates a vector space model (VSM) representation of the document corpus as well as the benchmark resource set. The VSM introduced by Salton et al [30] models documents as elements in term space. The term space is composed of all unique terms in the document collection and each document is represented by the vector of terms found in the document. Thereby the documents are comparable within the corpus and with external content. VSM has been successfully applied to several text mining/business analytics applications such as ontology-based information retrieval [31], incremental learning from text [32], and disease identification [33]. The VSM follows a term weighting scheme to improve the semantic position of a document. The 3 main factors of term weighting are term frequency factor, collection frequency factor, and length normalization factor. Term frequency factor determines the frequency within a single document, collection frequency factor determines its prevalence within the collection of documents, and the length of each document is used as a normalization factor to negate the bias of long documents.

A noted weakness of VSM is the assumption that identified terms are independent of each other. This shortcoming is offset to a certain degree with the inclusion of multi-word terms. Multi-word terms are able to capture more semantics than a single term set. The general VSM only focuses on single terms; therefore, it is necessary to create a separate VSM for multi-word terms. Thereby two VSMs *(vsm_m_(d_q_) , vsm(d_q_))* are created for each document *d*_*q*_ in each collection *D* generated by query *q*.

The VSMs generated for the document corpus need to be evaluated for relevance to the target audience and their information needs. Resources in the expert-endorsed content repository are the most suitable benchmark for this purpose. Independent to the VSMs from the document corpus *D*_*q*_, separate VSMs need to be generated for these resources in the content repository. The same query sent into the content search phase is run on the content repository to identify the relevant documents, *R_q_=r_1_,r,….r_n_ ∀q ∈Q*. The content of the documents in this set is converged into a single representative document and this is sent through to the multi-word term recognition phase followed by the generation of VSMs for both multi-word terms and single terms, *vsm_m_(R_q_)* and *vsm(R_q_)*, respectively. The outcome from this submodule is, for each query, a set of VSMs that represent new documents found in the content search phase and a set of VSMs that represent existing resources that are have been determined by the domain expert to be relevant to the same query. Effectively, this produces a benchmark term vector and the VSMs for multi-term words, *vsm_m_(R_q_)* and *vsm_m_(d_q_) ∀d∈Dq*, as well as for single terms *vsm(R_q_)* and *vsm_m_(d_q_) , ∀d∈D_q_*. Both these are defined using related dimensions that enable comparisons as well as rankings.

**Figure 8:**
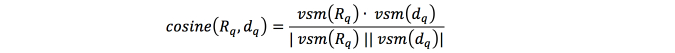
Calculation of cosine coefficient similarity measure.

The cosine coefficient similarity measure, which measures the angle between two vectors without bias for the length of the document, can be used to determine the closeness of each *d*_*q*_ to *R*_*q*_ (Figure 8). The denominator length-normalizes the vectors, ensuring the two are comparable in their original format. The same measure is calculated for the multi-term VSMs.

### Multi-Criteria Ranking

Thus far, the technique has generated 3 quantifiable measures: the ranking from content search, cosine similarity for multi-term words, and cosine similarity for single terms. Each measure represents an independent aspect of the content discovery process. The ranking from content search indicates the position assigned by the search engine (determined by the respective search and indexing algorithms) as well as its temporal significance. On the other hand, the cosine similarities are entirely content-based with the multi-term VSM capturing more semantics.

From a CM perspective, the quality of content is largely determined by 4 criteria; relevance, reliability, timeliness, and usefulness [8]. These can be defined briefly as relevance to the search query, usefulness to the target audience, reliability of the author/publishing body, and timeliness as the period when the article was compiled and published. As mentioned in the technique thus far, the existing content repository makes a significant contribution toward the relevance factor of new content. The content-based similarity measures are sound candidates for the measurement of relevance. Ranking from content search maintains temporal significance. This can be coupled with the actual date of publication, which can be retrieved from the host site (if available) to create a measure of timeliness. The author/publishing body of new content can be directly validated against authors/publishers of similar content found in the repository so that reliability can also be established to some extent. Usefulness that cannot be determined without user involvement/feedback is the only measure of quality that is beyond the proposed content discovery technique. The quality criteria are shown in Table 1.

**Table 1:** Means of quality measurement derived from the technique.

Quality criteria	Means of measurement
Relevance 1	Multi-word term similarity measure
Relevance 2	Single-word term similarity measure
Reliability	Direct validation of author/publishers with existing content
Timeliness	Content search ranking and date of publication
Usefulness	Not measurable (requires target audience involvement)

Multi-criteria decision-making (MCDM) involves the identification of an alternative from a finite set based on the evaluation of values from a set of criteria that characterize the alternative [34]. Ranking of new content is a variation of MCDM where more than one alternative is selected from a set of resources based on the assessment of four factors of quality. Several methods have been proposed to address MCDM problems: crisp methods such as multiplicative exponential weighting, simple additive weighting, analytic hierarchy process [35], discrete choice analysis [36], data envelopment analysis [37], and fuzzy MCDM analysis. Fuzzy MCDM analysis is largely based on the decision-making method in a fuzzy environment developed by Bellman and Zadeh [38]. The measures of quality will reflect varying degrees of importance for each ontology term. Given this subjective nature of the qualitative factors, it is pertinent to use fuzzy MCDM analysis for selection of new content.

An MCDM problem consists of 5 elements: alternatives, criteria, outcomes, preferences, and information [39]. In the context of content ranking, the alternatives are the new content discovered, the criteria are the measures of quality, preferences are the expectations for each criterion, and the quantified measures contain the information used to evaluate these parameters. The preferences, expectations for each criterion, are subjective because they vary between terms in the domain ontology. For instance, the measure of timeliness may not be as important as relevance for certain areas of the domain that are highly theoretical with less change over time. In such cases, the outcomes can be misleading if timeliness is equally represented as relevance in the ranking scheme. In essence, the criteria are sensitive to the type of term that is being evaluated. Fuzzy MCDM analysis is advanced to overcome this limitation. The advantage of using a fuzzy approach is in the assignment of relative importance of criteria using fuzzy numbers instead of crisp numbers.

**Figure 9:**
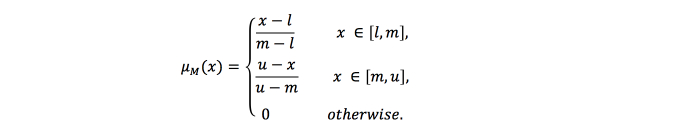
Calculation of membership function of M.

Fuzzy triangular numbers (FTN) are necessary to establish fuzzy weights for each criterion. Input provided by domain experts on the expectations of each criterion for each term is represented as FTNs. An FTN is defined as a fuzzy set, *F={(x,μ_F_(x),x∈R)*, where *x* takes values on the real line, *R:-∞ ≪x ≪ ∞* and *μ_F_(x)* is a continuous mapping from *R* to closed interval [0,1]. A FTN denoted as *M=(l,m,u),* where *l≪m≪u,* expresses the relative strengths of each pair of elements in the same hierarchy. The parameters *l; m; u*; represent the smallest possible value, the most promising value (modal), and the largest possible value respectively in a fuzzy event. The membership function of *M* is expressed as follows (Figure 9).

**Figure 10:**
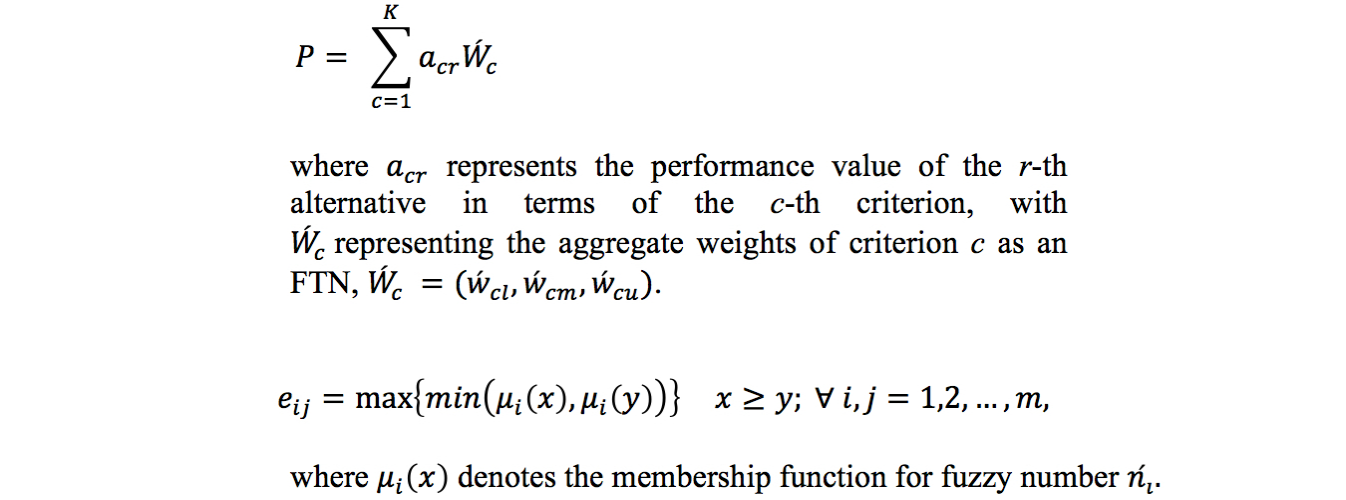
Calculation of derivation of weighted sum to measure performance (top equation). Once the weighted sum has been calculated, resources can be ranked (bottom equation).

The first 4 criteria (Table 1) relevance 1, relevance 2, reliability, and timeliness are defined as *C={c*_*1*_ *, c*_*2*_ *, c*_*3*_ *, c*_*4*_ *}* respectively. The weight of criterion *c* assigned to term *t* by expert *M*_*p*_ is denoted as FTN: *w^p^_c_*=(*l^p^_c_,m^p^_c_,u^p^_c_*), where *c ∈ {c*_*1*_ *, c*_*2*_ *, c*_*3*_ *, c*_*4*_ *}* and *p=*1,….*P*. The geometric mean is used to determine the aggregate weight when multiple experts provide input on expectations. The fuzzy score for criterion *c* of candidate resource *r* in terms of FTNs given by expert *M*_*p*_ is denoted as *s^p^_cr_=(LE^p^_cr_, ME^p^_cr_, UE^p^_cr_)* where *r=1,….m*, and *P*=1,….PP. An FTN for the weights of each criterion can thus be defined as *(m^p^_c_-ρ, m^p^_c_, m^p^_c_+ρ)*, where m^p^_c_ is the FTN mean and ρ is its spread, which is determined by domain experts and reflects the characteristics of criterion *c*. With *R* alternatives and *C* criteria, the weighted sum is derived to measure performance and shown in Figure 10.

Ranking takes place when *n_i_ > n_j_* if and only if *e_ij_*=1 and *e_ji_<Q*, where Q is a fixed position fraction of a number less than 1 (preferably 0.9). The use of a fuzzy MCDM approach has thus converted measures representing different qualitative factors into a single ranked metric based on weights indicative of the term from the domain ontology that is being explored by the technique. The ranked resources can now be easily perused by a domain expert.

## Results

As outlined earlier, SHIP was selected as the application test bed for the delineated technique. The technique was implemented using Java programming language for use in the experiments. Quality is essential for health information delivery and therefore maintenance and regular update of content is crucial for long-term value of the portal. The rate of generation of new health-related content far exceeds the numbers that can be manually examined by domain experts for relevance to a specific topic and audience. In this context, the benefits gained from the said technique are substantial. One of the portals noted earlier, BCKOnline, was used in this experiment. BCKOnline is a SHIP designed and developed at Monash University for the provision of personalized health information on breast cancer. A robust CM model was used by the domain experts to manage and revise the content in BCKOnline.

**Figure 11:**
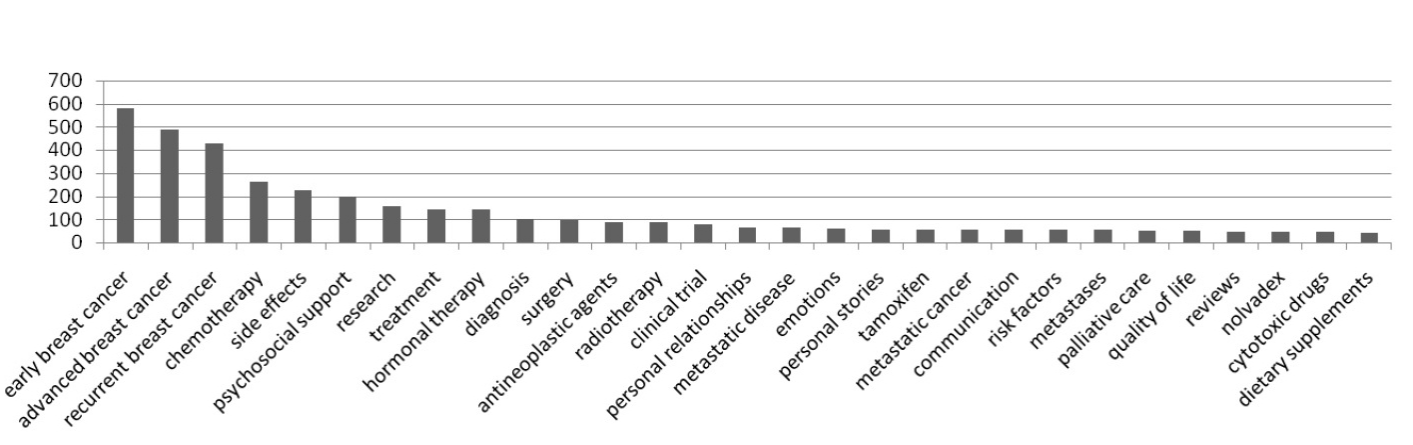
Top 30 domain ontology terms in BCKOnline.

The evaluation sample consisted of all content in the BCKOnline portal, a domain ontology comprising 795 terms and a content repository with 900 documents. Terms were selected from the ontology for demonstration of each phase. Each document was linked to one or more ontology terms by a domain expert. Figure 11 presents the top 30 domain ontology terms in the content repository. The graph exhibits a long tail, where a larger number of the resources are categorized in smaller groups. This signifies the breadth of health information for breast cancer accessible via the portal and further justifies the need for an automated content discovery process. The highest numbers of resources are on the primary subtopics of early, advanced, and recurrent breast cancer.

“Palliative care,” which has a count of 52 resources, was selected to demonstrate the query construction component. Construction of the query involves generic and specific dimensions (Figure 3). The actual term is the specific ontology dimension and the term “breast cancer” represents the high-level domain and its inclusion introduces a background to the query. The next level of construction expands the query to include personalization and diversification efforts. The audience dimension is represented using several attributes specific to the high-level domain of breast cancer. These are level of knowledge, age groups, stage of illness, and user role. WordNet is explored in search of the generic dimension of synonyms. The two terms, “palliative” and “care” are searched separately. The WordNet senses metric is used to select synonyms with a higher relevance to the input term. The association of dimensions for the said term is tabulated in Table 2. Starting with the base query “*palliative care breast cancer*,” the search is gradually expanded to include the audience attributes and the synonyms. Thereby, the recurrent phases of query construction and content search contribute toward good coverage of available online content.

**Table 2:** Dimensions of query construction for term “palliative care.”

Dimension	Values
Specific: audience	(basic, scientific, experiences), (young, middle-aged, old), (early, recurrent, advanced stages), (friend, partner, child)
Specific: domain ontology	palliative care
Generic: high level domain	breast cancer, breast carcinoma
Generic: synonyms: palliative	Directly related: alleviative, preventative, lenitive Inherited from: curative, remedial, therapeutic
Generic: synonyms: care	Directly related: aid, attention, tending Inherited from: work, action, procedure

After the search results have been processed into a corpus of plain text documents, *D_q_=d_q1_, d_q2_, …d_qn_*, multi-term recognition takes place. As mentioned earlier, this module identifies multi-word terms that are ignored by the VSM. The expectation of text analytics phase is to capture semantics representative of the documents; the inclusion of multi-word and single-word terms reinforces the VSM outcomes. As an illustrative example, some comparable multi-word terms and single-word terms recognized from a high ranked resource are presented in Table 3.

**Table 3:** Comparison of multi-word and single-word terms from an online resource on “palliative care”[40].

Multi-word terms	Single-word terms
palliative care, palliative care team, palliative care specialist, palliative medicine, anticipate future issue, spiritual care, outpatient setting, treatment option, family member	palliative, care, specialist, treatment, disease, female, support, family, body, medicine

**Figure 12:**
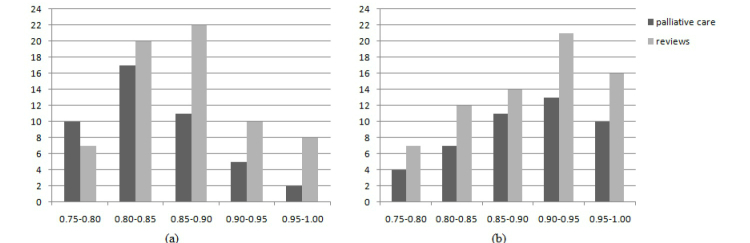
Histograms of similarities of new resources to benchmark VSM (a) single-word (b) multi-word terms.

In the term vector creation stage, VSMs for multi-term words, *vsm_m_(R_q_)* and *vsm_m_(d_q_) ∀d∈Dq*, as well as for single terms *vsm(R_q_)* and *vsm_m_(d_q_)*, *∀d∈Dq* are generated. Vector *R_q_* represents the benchmark vector derived from existing resources in the content repository. The cosine similarity was used to measure likeness between the VSMs with the threshold set at 0.75. Two terms were selected to demonstrate the measures of similarities. These are “palliative care” and “reviews.” The contrasting nature of the terms, the first being specific and the second more general, appeals to the usual content discovery requirements of information portal and related Internet technologies. The number of new resources above the threshold for the first term was 45 and 70 for the second term. The second term, “reviews” has a larger number of resources because it covers a broad content area. The cosine similarities in the range of 0.75-1 in bins of 0.05 are depicted in the histograms in Figure 12 for the multi-word and single-word VSMs of the two terms.

The primary observation here is the high similarities of most resources in the multi-word VSM, with 60 resources (23 for palliative care and 37 for reviews) in the range of 0.9-1.0 in comparison to single-word terms that have only 25 in the same range. This proximity to the benchmark is indicative of the contextual information captured by multi-word terms.

Multi-criteria ranking aims to satisfy 3 criteria: relevance, reliability, and timeliness. The multi-word and single word similarity measures make up 2 relevance measures. The ranking from the content search is coupled with the upload date and time of each resource to calculate a timeliness measure. Reliability is determined by comparing the author/publisher names of new resources with those already in the repository. Unknown authors are ranked very low so that domain experts can intervene at the actual content selection phase to determine reliability based on their knowledge. As already presented, the varying level of importance of criteria for each term prompted the use of fuzzy weights per criterion per term. Inputs accumulated from domain experts are accumulated and aggregated to generate these FTNs. The following FTNs (Table 4) were used for the 2 terms “palliative care” and “reviews” to demonstrate the multi-criteria ranking process. Both terms have high weights for the 2 relevance measures and reliability in contrast to timeliness. Timeliness is not crucial for the term “reviews” due to the obvious nature of a medical review. The reliability measure for “review” is weighted above that for “palliative care.” The weighted sum value, *a_cr_W_c_* for three resources for term “reviews” is presented in Table 5. The 4 measures for each resource were normalized to 1-10 and are shown in the first column of Table 5.

**Table 4:** FTNs used for ranking criteria.

Term	Relevance 1	Relevance 2	Timeliness	Reliability
Palliative care	(0.50, 0.70, 0.90)	(0.30, 0.50, 0.70)	(0.40, 0.60, 0.70)	(0.40, 0.50, 0.60)
Reviews	(0.60, 0.70, 0.90)	(0.60, 0.70, 0.90)	(0.10, 0.30, 0.40)	(0.40, 0.60, 0.90)

**Table 5:** Weighted measures for three resources for term “review.”

Resource name and measures	Relevance 1 (0.60, 0.70, 0.90)	Relevance 2 (0.60, 0.70, 0.90)	Timeliness (0.10, 0.30, 0.40)	Reliability (0.40, 0.60, 0.90)
R1 (7.50, 5.50, 4.10, 6.90)	(4.50, 5.25, 6.75)	(3.30, 3.85, 4.95)	(0.41, 1.23, 1.64)	(2.76, 4.14, 6.21)
R2 (5.40, 9.20, 8.70, 0)	(3.24, 3.78, 4.86)	(5.52, 6.44, 8.28)	(0.81, 2.43, 3.24)	(0,0,0,0)
R3 (8.50, 4.70, 6.80, 7.20)	(5.1, 5.95, 7.65)	(2.82, 3.29, 4.23)	(0.68, 2.04, 2.72)	(2.88, 4.32, 6.48)

**Figure 13:**
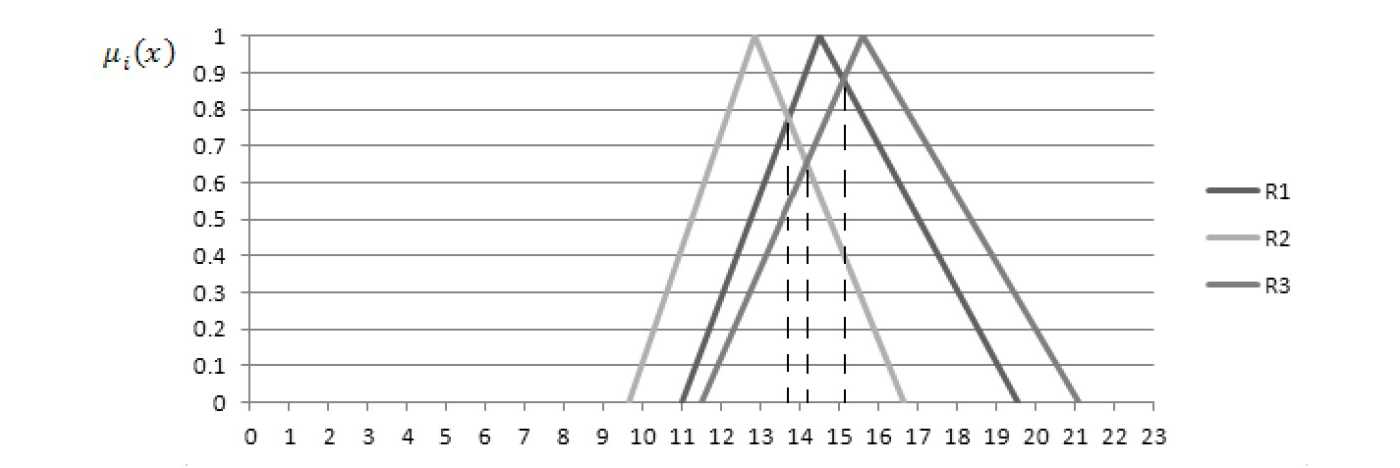
Membership functions for weighted summations of R1, R2 and R3 metrics.

The weighted summation of the resources are R1 (10.97, 14.47, 19.55), R2 (9.63, 12.83, 16.62), and R3 (11.48, 15.6, 21.08). Figure 13 displays the membership functions for each. Following Figure 10, the comparison scores are *e_31_, e_32_, e_12_*=1, *e_13_*=0.88, *e_21_*=0.76 and *e_23_*=0.64. Using a threshold *Q* of 0.9 and 0.8, respectively, the ranking of the 3 resources in descending order can be determined as R3, R1,and R2. With completion of the ranking phase, the ranked resources and the intermediary metrics are sent through to the domain expert for further scrutiny.

## Discussion

Evaluation and quality of content become crucial based on the information expectations of the target audience, especially in the case of health information [1]. The increase in relevant online health information is a challenge for domain experts to peruse and evaluate on a regular basis. This paper reported the development of an intelligent content discovery technique that is able to address this challenge with automated discovery and ranking features. The technique utilizes an existing content repository as a benchmark to validate new content discovered online. It operates in 4 modules: query construction, content search, text analytics, and multi-criteria ranking. Query construction uses an existing ontology of key terms and supplements this with audience and context information as well as synonyms extracted from WordNet. Content search retrieves a unique list of resources that are downloaded, preprocessed, and consumed by text analytics. Semantics, based on multi-word and single-word terms, are identified in text analytics and used to measure proximity to a benchmark vector derived from existing content. Acknowledging the subjective nature of qualitative factors, fuzzy weights are used in the multi-criteria ranking phase to determine a single rank encompassing relevance, reliability, and timeliness. The paper delineates the complete technique with an inclusive demonstration of its execution using an actual health information portal as a test bed. The technique can be sufficiently generalized and applied in other domains. In the next phase of the project, we will focus on validation of the technique with experiments involving domain experts as well as user studies to highlight its benefits and further establish its purpose in CM. Future research will also investigate the advantages of ripple-down rules [41] over fuzzy MCDM when generalizing the technique for application in other domains with incremental usage over time.

## Abbreviations

CM: content management
FTN: fuzzy triangular numbers
MCDM: multi-criteria decision-making
SHIPs: smart health information portals
VSM: vector space model
